# RSV awareness, risk perception, causes, and terms: Perspectives of pregnant and lactating women in Kenya to inform demand generation efforts for maternal RSV vaccines

**DOI:** 10.1080/21645515.2023.2258580

**Published:** 2023-10-09

**Authors:** Rupali J. Limaye, Berhaun Fesshaye, Prachi Singh, Ruth A. Karron

**Affiliations:** a International Vaccine Access Center, Johns Hopkins Bloomberg School of Public Health, Baltimore, MD, USA; b Department of International Health, Johns Hopkins Bloomberg School of Public Health, Baltimore, MD, USA; c Department of Epidemiology, Johns Hopkins Bloomberg School of Public Health, Baltimore, MD, USA; d Department of Health, Behavior & Society, Johns Hopkins Bloomberg School of Public Health, Baltimore, MD, USA; e Center for Immunization Research, Johns Hopkins Bloomberg School of Public Health, Baltimore, MD, USA

**Keywords:** Maternal immunization, RSV, Kenya, pregnant persons, lactating persons

## Abstract

Respiratory syncytial virus (RSV) causes a substantial proportion of acute lower respiratory tract infections (LRTI) among infants. In low- and middle-income countries, RSV may be responsible for approximately 40% of all hospital admissions of infants less than one year. A safe and immunogenic RSV vaccine, given to pregnant persons, is imminent. In this qualitative study, we sought to understand factors that could inform maternal vaccine decision-making to inform future demand generation strategies in Kenya. We conducted in-depth interviews with 24 pregnant and lactating persons from two counties, with two communities in each county. Four key themes emerged, including terms used for RSV, awareness of and risk perception related to RSV, causes of RSV, and questions about future maternal RSV vaccines. Regarding terms, no participant used the term RSV to describe the disease. Most participants associated RSV with cold things such as cold weather and cold food/drink. Most participants believed that RSV was caused by the cold or an unclean environment. Finally, key questions related to a maternal RSV vaccine were related to vaccine safety, and more specifically side effects. Questions arose related to vaccine effectiveness as well as timing of administration and dosing. A maternal RSV vaccine is on the horizon. However, vaccines do not save lives; vaccination does. As such, it is critical to develop and implement demand generation approaches to ensure that once a maternal RSV vaccine is available, communities are sensitized and willing to accept it.

## Introduction

Respiratory syncytial virus (RSV) is a significant cause of acute lower respiratory tract infection (LRTI) among infants globally.^1^ In sub-Saharan Africa and Asia, RSV may be responsible for approximately 40% of all hospital admissions with severe or very severe pneumonia among infants under 1 year.^2^ Severe RSV-associated LRTI is most common among infants under six months of age.^3^ In most healthy adults, RSV is mild; RSV infection in pregnant women is variable, ranging from mild disease to a more severe illness.^4^

The greatest burden of severe RSV disease is during the first 3 months of life.^3^ As such, maternal immunization is one key strategy to protect young infants.^5^ Based on the principle of active transplacental transport of IgG antibody across the placenta, maternal immunization can protect the infant against RSV disease during their most vulnerable period. Maternal RSV immunization is an attractive preventative strategy to reduce maternal and neonatal morbidities and mortalities. Efforts to develop a maternal vaccine to prevent RSV disease in infants are far advanced: as of this writing, the Pfizer RSVpreF vaccine has completed phase 3 evaluation and is under review for licensure/market authorization by regulatory authorities.^6^

Several studies have estimated disease burden and cost-effectiveness of an RSV vaccine program.^7,8^ For example, a mathematical model demonstrated the potential effect of a maternal RSV vaccine, reporting a likely reduction in rates of hospitalization for RSV by 6–37% for infants under three months of age, and 30–46% for children between three and five months of age, depending on the level of vaccine efficacy.^9^

While studies have been able to articulate potential effects of a maternal RSV vaccine, these effects can only be realized if individuals accept the vaccine. A safe and immunogenic RSV vaccine, given to pregnant persons during the second or third trimester, may become globally available within the next few years. However, product availability does not necessarily translate into successful uptake and acceptance. Creating and sustaining demand for immunization services is essential to ensure that vaccine-eligible populations are fully protected from vaccine-preventable diseases.^10^ In this study, given the burden of RSV disease in Kenya we sought to understand factors that could influence the decision-making process for maternal RSV vaccine acceptance in pregnant and lactating women in Kenya to inform future demand generation strategies.^11^

We focused on both pregnant and lactating women because while a maternal vaccine would be given in pregnancy, given that the disease primarily affects infants, understanding attitudes of pregnant as well as lactating women will need to be taken into consideration for successful vaccine acceptance.

## Methods

This qualitative study conducted in-depth interviews with pregnant persons and lactating persons. Participants were recruited from two counties, with two communities in each county: Nakuru (rural), and Mombasa (urban). Participants were recruited at health facilities that had antenatal care clinics across the four communities. Ten sites in each county (18 public hospitals and 2 private hospitals) were used for recruitment, representing Level 1 through Level 5 facilities. Facilities were chosen based on level type and location, as well as client flow. We sought to enroll the same numbers of participants across the 10 facilities. Research assistants recruited participants at a specific clinic until the enrollment target was met. At each clinic, a research assistant would be present in the waiting room of antenatal clinics during the clinic’s hours of operation. All women that were in waiting rooms of antenatal care clinics were approached by a research assistant to enroll in the study.

Data were collected in August-September 2022. Data collectors participated in a three-day training exercise and completed an online human ethics training. At each clinic, after a research assistant explained the purpose of the study and a participant was interested in being a part of the study, participant eligibility was ascertained. If a participant met the inclusion criteria [at least 18 years of age, able to give consent, pregnant (past 1st trimester), or lactating] and agreed to participate, oral consent was obtained. Interviews were conducted in English, Swahili, or other local languages as necessary in a semi-private setting. All interviews were audio recorded, then later transcribed, and translated into English by members of the study team and external translators fluent in both languages. All data, including audio recordings, were stored on encrypted servers, and only members of the study team had access to the data. Interview guides were pre-tested with pregnant persons living in Kenya. The guide included questions related to awareness of and experiences with RSV, risk perception related to RSV, causes of RSV, and questions related future RSV vaccines.

A team of 6 used a grounded theory approach to analyze the data. Data were managed using Atlas.ti. The code list was developed, refined, and finalized over three rounds of open coding. Following agreement of a code list, the team coded the transcripts, holding discussions on emerging themes after coding 50% of the transcripts. Two members of the team conducted inter-rater reliability with ~ 10% of the transcripts that neither of them had coded (3 transcripts). Reliability was 89%. The team then identified themes and sub-themes. This study received ethical approval from the Kenya Medical Research Institute and the Institutional Review Board of the Johns Hopkins Bloomberg School of Public Health (IRB00014893).

## Results

**Figure 1.**
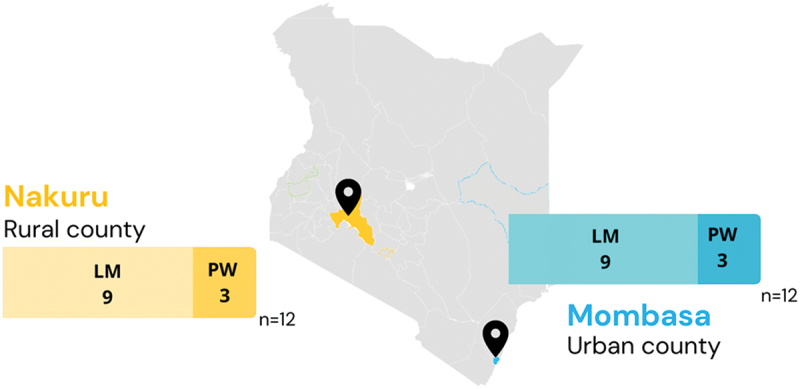
Map of sampled populations and locations across Kenya (*n* = 24 interviews; 18 lactating mothers, 6 pregnant women).

A total of 24 persons were interviewed, with 18 lactating women and 6 pregnant women (Figure 1). All identified as women. Half of each participant type were from rural communities, and half were from urban settings. To explore participants’ familiarity with the clinical presentation of RSV, we began each interview with a short, 10-second video of a baby with RSV that had RSV-characteristic wheezing. Several themes emerged from the interviews related to generating demand for future maternal RSV vaccines, including terms used for RSV, awareness of and risk perception related to RSV, causes of RSV, and questions about future maternal RSV vaccines.

### Terms used for RSV

We wanted to understand if pregnant and lactating persons were aware of RSV, including whether or not they knew someone who had experienced RSV in their family or community. When asked if they had heard of RSV, none of the participants said they had heard of it. After showing the short video at the start of the interview, interviewers informed participants that the video showed a baby with RSV, and then asked participants what terms they used to describe RSV. Table 1 describes all of the various terms used by participants for RSV. The most common term used was pneumonia, as well as flu, pumu, and limonia.

**Table 1.** Terms used to describe RSV by pregnant women and lactating mothers.

	Pregnant women (*n* = 6)	Lactating mothers (*n* = 18)
*Terms used by two or more participants*	Pneumonia (*n* = 2)	Pneumonia (*n* = 11) Flu (*n* = 4) Pumu (asthma) (*n* = 2) Limonia (a living thing) (*n* = 2)
*Terms used by one participant*	Asthma Breathing hard Cold Kirithigi (flu of the lungs) Wheezing in the heart	Chest congestion Cold Malaria Kukhera (wheezing)

### Awareness of and risk perception related to RSV

After showing the participants the video and informing participants that what they saw as a baby with RSV, we then asked questions related to awareness of RSV in their communities and who the disease affected, to understand their risk perception. After seeing the video, most participants said that they knew what RSV was. We asked participants to describe RSV, and participants commonly described RSV as a cold that caused difficulty in breathing in younger children, as described by this lactating woman: “*It makes the baby to have difficulty in breathing and cries and having running nose. You know when they are small, they get cold easily maybe when they reach like two years – the disease disappears”* (Lactating woman, Kamara Dispensary, Nakuru). Many participants said it was common among younger children because of their immune systems, including this pregnant woman: “*It is common in infants because they are so young, and their immune system has not developed, hence they are very prone to getting that disease*” (Pregnant woman, Mombasa Hospital, Mombasa). According to participants, the underdeveloped immune system of young children was also the reason why younger children were more likely to get severe disease, as articulated by this lactating woman: “*Small children are the ones who get it often and easily get worse – their immunity is low*” (Lactating women, Kiptangwanyi Health Centre, Nakuru).

Besides the agreement that younger children were more susceptible to RSV, almost every participant was able to identify RSV because of wheezing, as this lactating woman commented: “*In fact, my sister’s child, she had pneumonia and had symptoms – that difficulty in breathing*” (Lactating woman, Mlaleo Health Centre, Mombasa). This lactating woman mentioned the severity of RSV: “*The child was … had difficulty in breathing. The child refuses to breastfeed, had a lot of mucus. It was serious because the child refused to eat and to breastfeed. And when we see the child is refusing to eat, you know that child is very sick*” (Lactating woman, Bahati Rural Health Centre, Nakuru). Similarly, this lactating woman also referred to the wheezing symptom as indicative of a severe disease: “*It can reach a time when you are defeated to breathe, you feel your chest burning, you feel like you are being stabbed in the chest when you are breathing and also there is that, wheeze, that wheezing sound, and you are coughing continuously until you feel pain in the chest*” (Lactating woman, Tudor Sub-County Hospital, Mombasa).

Due to the belief that younger children were more susceptible to RSV, many parents believed that it was important to protect the baby during the cold season, when children were more susceptible, as described by this pregnant woman: “*From the month of April up to December – that is when those are the colder months and there is that disease*” (Pregnant woman, Bahati Rural Health Centre, Nakuru). As such, participants spoke frequently about how keeping a baby warm would make their child less susceptible to RSV, as described by this lactating woman: “*My, child … no I do not see him getting RSV because I am very careful when it is cold, I keep him warm. So, the probability of him getting that disease is very low*” (Lactating woman, Nakuru Level 6 Hospital, Nakuru). This pregnant woman also talked about dressing the child warmly for protection against RSV: “*They might get because now it is the rainy season and you might find the child has gone somewhere and you have not been able to dress the baby warmly, so they can get it*” (Pregnant woman, Molo Sub-County Hospital, Nakuru).

Related to experience with RSV in their communities, participants mentioned that parents often delayed seeking care for their child that had that disease, because they dismissed it as a cold, per this lactating woman: “*I have seen some children breathe like that, but they often dismiss it as cold. They are given medicine and it eventually stops. I have seen some neighbors’ child that was very young and was breathing with difficulty, but the child did not last. At night the infant was taken to hospital and the breathing even got worse, on the second day, the child passed away. I have seen that with my own eyes. On the second day, the child passed away and we buried him*” (Lactating woman, Mrima Health Centre, Mombasa). Another lactating woman also mentioned the delay in seeking care: “*When the child is refusing to eat, and the child is breathing like wheezing, it is a problem because you may delay taking the child to the hospital and the child dies*” (Lactating woman, Bahati Rural Health Centre, Nakuru). This pregnant woman also spoke of challenges in obtaining treatment for RSV: “*It is a problem because when you have that disease of respiratory … you are having an attack at night, and a night you have you no means, you have no money because that is something sudden. So, (RSV) can give challenges in terms of treatment*” (Pregnant woman, Port Reitz Sub-County Hospital).

### Causes of RSV

We asked about the causes of RSV, to better understand if the participants understood that a virus causes RSV. The cold and effects of the cold was the most common cause given, with an unclean environment the next most common cause.

Both pregnant women and lactating women believed that not dressing warmly – by both the mother and the child – could cause RSV. This lactating woman noted the importance of the mother dressing warmly: “*You know it can be caused by the mother, maybe the mother is not dressed warmly and goes out into the rain and then when she comes back, she starts breastfeeding the baby while she is still wet. If you do not dress the baby warmly the baby will feel cold. Also, wetness – when they touch water it can infect the baby with RSV*” (Lactating woman, Kamara Dispensary, Nakuru). Another lactating mother noted the importance of dressing a baby warmly: “*I usually tell someone a baby, even if he is crawling, he does not know about heat. You see the baby is sweating, but if you cover the baby with clothes properly or you protect the baby, they cannot be infected with any illness like that one of pneumonia, that one of congested chest, they cannot get it. Maybe they can get malaria. As for me I can say it is during hot seasons because during hot seasons you see the baby sweating and you dress them warmly, it is like you are punishing the baby. But you dress them warmly or you undress them because a baby gets rashes, it is better for them to have rashes than being infected with that illness*” (Lactating woman, Jomvu Model Health Centre, Mombasa). This lactating woman also mentioned dressing the baby warmly during the cold season: “*During the cold seasons when you bathe a baby and do not cover them and also if you do not dress them warmly you can get that disease*” (Lactating mother, Magongo Dispensary, Mombasa).

Some women indicated that the effects of cold – such as dampness, for example, caused RSV, including this lactating woman: “*When you take cold things a lot, it can be cause of being infected with that illness, sometimes also it is cold, there is that normal cold and there is also that cold, how do they say, damp cold, like when something stays in a place for long. It becomes damp. You find a person covering the baby at the time it is very hot and now those clothes become wet with sweat, but the mother does not remove them, she leaves them on them until they get dry on the baby, so that also causes that disease*” (Lactating woman, Tudor Sub-County Hospital, Mombasa). This lactating woman also believed dampness caused RSV: “*The problem with our place is that here from evening it is normal until 12.00 a.m … then it is hot, the children are sweating, then from 2am to 3am it becomes cold. This contributes because as a parent you have forgotten and the baby is sweating, so during that time you do not remember to remove the clothes which are wet with the sweat. So that wetness with that colder air – I think that can cause the virus*” (Lactating women, Junda Dispensary, Mombasa).

Other women pointed to consuming cold food or drinks as causing RSV. This lactating woman believed her consumption of cold water during her pregnancy caused her child to get RSV: “*That disease infected my second born child. I passed through challenges because I did not know how I could handle him because he used to get congested until I brought him to the hospital, and he was given oxygen and he looked like he was going to leave me. But what I came to realize in my own opinion, I thought it was the cold water which I used to drink while I was pregnant because I used to like cold water*” (Lactating woman, Port Reitz Sub-County Hospital, Mombasa). Another lactating woman pointed to the child being exposed to cold water as a cause: “*Yes, I have seen that disease in a child who was about eight months old. The child’s mother exposed the child to cold water and the mother did not have him wear shoes or sock. The cold got into this child*” (Lactating woman, Likoni Sub-County Hospital, Mombasa). This lactating woman pointed to giving the baby cold food as the cause: “*When you give them cold food, it can cause things like that virus*” (Lactating woman, Kamara Dispensary, Nakuru). Similarly, this lactating woman referred to the consumption of cold things while pregnant as a cause of RSV: “*Like drinking cold water, drinking cold things, you know those cold things you drink them when you feel hot in your body, but it will affect a place which does not know what the heat is. The cold affects you because you were drinking cold drinks from one month until nine months so how much cold have you added there? It is a lot so by the time you are giving birth to the baby, the baby must be infected with that virus*” (Lactating woman, Port Reitz Sub-County Hospital, Mombasa).

Several women believed that an unclean environment caused RSV. For example, this lactating woman referred to dirt and wind: “*I do not know what causes this disease but I feel it is caused by dirty environment, when you are using the same things communally like someone is having a bad sweats and you are using the same clothes or you also wear their clothes, things like that or house utensils when you are touching them and using them together, so if one is infected and you are using these things together you will also get infected because sometimes you do not know when they touched the things, so you will be infected as well*” (Lactating woman, Miritini Health Centre, Mombasa). This lactating woman referred to unclean food and an unclean house as a cause: “*Maybe the food that the child eats, meaning it could be food that is not useful. If the child eats rice, it may not be useful. So, I can say it’s the food the child uses*” (Lactating woman, Rongai Health Centre, Nakuru). Finally, this lactating woman believed RSV was caused by the outside environment: “*This pumu disease … I feel it is the air or it is the dirt. When I talk about air, I mean like maybe a certain wind comes and passes by, so there are those things and that wind and dirt*” (Lactating woman, Miritini Health Centre, Mombasa).

### Questions related to maternal RSV vaccines

We wanted to understand if an RSV vaccine was available and approved for pregnant persons, what questions participants would have about the vaccine. We asked participants to consider a hypothetical situation where a maternal RSV vaccine was available and was approved for use in pregnant women by the Ministry of Health and then think about what questions they would have about this vaccine. The most common questions that arose were related to vaccine safety, and more specifically side effects. Participants also had questions about vaccine effectiveness, and timing of administration and dosing.

Many women had questions about potential side effects of the vaccine. This lactating woman was concerned that the vaccine could affect the baby: “*That vaccines can … that the baby can be affected, and the mother can be affected by vaccines. That they have a miscarriage, or they may get sick*” (Lactating woman, Rongai Health Centre, Nakuru). This lactating woman mentioned that an understanding of side effects would be the reason why she would accept the vaccine: “*I would ask if it can affect someone and if they tell me it does not affect then I would accept to be injected*” (Lactating woman, Mwisho wa Lami Dispensary, Nakuru). This lactating woman was concerned that the vaccine could affect employment: “*Maybe if I am injected, maybe I can get ill, after being vaccinated, maybe I will be unable to work. But all that you can’t tell*” (Lactating woman, Nakuru Level 6 Hospital, Nakuru).

In addition to potential side effects, many women also wanted to better understand the effectiveness of the vaccine and how the vaccine would protect against RSV. This pregnant woman said: “*I would like to know how that vaccine is going to work in my body and how it will go and work on the baby’s body because now it still in the womb and can it have what, like any side-effects which can affect a person or the baby, now things like those*” (Pregnant woman, Bahati Rural Health Centre, Nakuru). This lactating woman was also interested in understanding the effectiveness of the vaccine: “*I would ask what the vaccine help with. I would ask before receiving the vaccine*” (Lactating woman, Likoni Sub-County Hospital, Mombasa). This lactating woman also mentioned effectiveness for whom: “*I would want to know if it can affect the mother, or if it will also affect the child who the mother is carrying*” (Lactating woman, Bahati Rural Health Centre, Nakuru). This pregnant woman was curious about benefits to the baby after it was born: “*I would want to know the benefits of having the vaccine, how will it benefit maybe my kid, the baby for the pregnant mother being vaccinated. After birth – I wonder if, how will it benefit the baby maybe after birth*” (Pregnant woman, Mediheal Hospital, Nakuru). Similarly related, there were questions about whether or not only pregnant women would receive the vaccine: “*And now when a baby is born they cannot be injected on their own? But if you are not injected when you were pregnant when you get a baby will you be injected*?” (Lactating woman, Mwisho wa Lami Dispensary, Nakuru).

Related to effectiveness, timing was another question that frequently arose. This lactating woman wondered about protection of the vaccine after delivery: “*So on my part, maybe let us say I have already given birth, so what will be the use of me getting that vaccine and the person who it is supposed to protect is this one who is outside now*” (Lactating woman, Port Reitz Sub-County Hospital, Mombasa). This pregnant woman wondered about dosing: “*You must have questions because you feel, how does it work and what is its duration, can it that it will be given only once or every time you come to the clinic you are injected, you come again next visit, or you are injected only once*” (Pregnant woman, Port Reitz Sub-County Hospital, Mombasa).

## Discussion

Few studies have examined RSV awareness among pregnant and lactating women. Only after showing participants the video of the baby with characteristic RSV wheezing and explaining that what the baby had was RSV, participants informed us that they were familiar with the disease. This finding was not congruent with the only other study we could find that assessed RSV awareness among pregnant women; however, this study was conducted in Australia^12^ which is a very different context. However, none of our participants used the term RSV itself to describe RSV. This is line with another study that examined perceptions of RSV and found that pregnant women and health care providers used terms such as bronchiolitis to describe RSV, indicating the need to think about how to promote RSV prevention tools, including maternal immunization.^13^ Results from our study indicate that when a maternal RSV vaccine is available, demand generation efforts should consider how to promote and communicate about an RSV vaccine, including what to call the vaccine, as participants in our study did not use the term RSV when talking about RSV. This also includes ensuring that pregnant women understand the disease the vaccine would prevent – as mentioned, showing women a video of a wheezing baby helped to ensure that when we asked participants about RSV, they were providing perspectives on the disease we were interested in understanding.

Study results suggest that few people understand how RSV is transmitted, as not one study participant was able to correctly identify the cause of RSV. Given that most participants believed that RSV was caused by cold weather and an unclean environment, it will be imperative that RSV vaccine demand generation efforts include ways to best convey that RSV is caused by a virus, and the ways in which the RSV vaccine will interact with the virus that causes it.

Participants had questions related to maternal RSV vaccines that would need to be answered before accepting vaccines, and most of these questions focused on vaccine safety broadly, and side effects more specifically. As we were able to ascertain specific questions related to maternal RSV vaccines, these questions can be used to inform demand generation materials targeted toward pregnant women, to be responsive to their questions. It is not surprising that the overriding factor driving a woman’s decision to be immunized during pregnancy is safety. This is in line with other studies exploring the vaccine decision-making process among pregnant women in Kenya.^14,15^

Related to vaccine safety of vaccines given during pregnancy, a systematic review that examined the vaccine decision-making process among pregnant women related to seasonal or pandemic influenza found that beliefs that the vaccine would cause no harm to the pregnancy were strongly associated with vaccine acceptance, as beliefs that the vaccine could cause birth defects or general harm in pregnancy were strong deterrents to accepting maternal vaccines.^16^ An integrative review found that the optimal time to provide education and information to inform vaccine decision-making is during pregnancy, indicating the essential role health care providers play in information provision.^17^ Given the questions that arose from our study participants, highlighting the benefit of the vaccine and clearly stating any potential harms related to the vaccine should be discussed during pregnancy with a trusted health care provider. Explaining timing of the vaccine – given during pregnancy and not after childbirth – will also be critical to communicate and ensure women understand the delivery, given the questions that arose from study participants in relation to timing.

This study has limitations. This qualitative study was not designed to be generalizable, and social desirability bias is likely. Findings were heavily dependent on the cross-sectional nature of the study. This study also has strengths. Little is known about vaccine intentions among pregnant and lactating persons related to future RSV maternal vaccines. Understanding the perspectives of this population will be crucial to create demand for new maternal vaccines, including the RSV vaccine, and affect subsequent acceptance. Additional research is needed to better understand other critical decision-making constructs among pregnant and lactating women, as these vary by location, context, and target population. Given that vaccine decision-making is complex, there are many other constructs that should be explored in future studies.

Maternal immunization can substantially reduce the burden of infectious diseases in mothers and infants. But for this approach to be realized and successful, communication to the public for demand generation is essential, specifically communication that understands and addresses the concerns of pregnant persons. There is operational work which can be started to ensure successful implementation if a safe, effective maternal RSV vaccine becomes available in the future.^18^ This includes raising stakeholder awareness of RSV disease and maternal immunization.^4^ Preemptively ascertaining the level of awareness of RSV among pregnant people may allow public health practitioners to identify interventions to optimize future uptake. The findings from our study – related to awareness, risk perception, causes of RSV, and questions related to a future maternal RSV vaccine – can directly inform demand generation and communication materials once a maternal RSV vaccine is available.

A maternal RSV vaccine has the potential to decrease all-cause pneumonia as well as RSV-specific disease and to have long-term effects on pediatric lung health.^19,20^ Given that a maternal RSV vaccine candidate may receive regulatory approval in 2023 and become globally available within the next few years, it is increasingly important to plan for introduction and successful delivery. However, vaccines do not save lives; vaccination does. We need to be able to communicate the value proposition of maternal immunization.^21^ This includes counseling on the benefits and safety of a vaccine that emphasize the vaccine’s protective effect on the pregnancy and clearly articulate implications for fetal and childhood development.^16^ Given this, being able to effectively communicate and promote acceptance and uptake will be critical for the benefits of such vaccines to be realized at both individual and societal levels. While we acknowledge that additional work is needed to nudge pregnant and lactating women toward future maternal vaccine acceptance, we believe our study results are a step in this direction.
